# Mitochondria–endoplasmic reticulum contact sites in hepatocytic senescence

**DOI:** 10.1186/s11658-025-00809-4

**Published:** 2026-01-11

**Authors:** Pavitra Kumar, Mohsin Hassan, Frank Tacke, Cornelius Engelmann

**Affiliations:** 1 Department of Hepatology and Gastroenterology, Medizinische Klinik M. S. Hepatologie und Gastroenterologie, Charité Universitätsmedizin Berlin - Campus Virchow-Klinikum, Augustenburger Platz 1, 13353 Berlin, Germany; 2 Berlin Institute of Health (BIH), 10178 Berlin, Germany

**Keywords:** Mitochondria, ER, Contact sites, MERCs, Calcium, Hepatocyte, Senescence

## Abstract

Inter-organelle communication via membrane contact sites (MCSs) is essential for the efficient functioning of eukaryotic cells, facilitating coordination among approximately 20 distinct organelles, each with unique metabolic profiles. Among these interactions, mitochondria–endoplasmic reticulum (ER) contacts (MERCs) are particularly significant, encompassing about 5% of the mitochondrial surface. Key proteins involved in MERCs include inositol 1,4,5-trisphosphate receptor (IP3R), voltage-dependent anion channel (VDAC), glucose-regulated protein 75 (GRP75), Sigma1 receptor (Sig-1R), vesicle-associated membrane protein (VAMP)-associated protein B (VAPB), protein deglycase DJ-1, and protein tyrosine phosphatase interacting protein 51 (PTPIP51), with new proteins continually being identified for their roles in these structures. At these contact sites, metabolic exchanges involve calcium (Ca^2+^), lipids, reactive oxygen species (ROS), and proteins. MERCs enable efficient molecular exchanges through temporary bridges mainly formed by the ER, the organelle with the largest surface area. These contacts are crucial for maintaining mitochondrial dynamics, which is essential for cellular homeostasis, and they are notably impacted in pathological states such as metabolic dysfunction-associated steatotic liver disease (MASLD), alcohol-related liver diseases (ALD), and viral hepatitis. Dysfunctional MERCs can lead to mitochondrial fragmentation, increased ROS production, impaired autophagy, and disrupted protein trafficking, thereby exacerbating senescence and cellular aging. Senescence is a cell fate initiated by stress, characterized by stable cell-cycle arrest and a hypersecretory state, and is an underlying cause of aging and many chronic conditions, including liver diseases. The hallmarks of senescence—such as macromolecular damage, cell cycle withdrawal, deregulated metabolism, and a secretory phenotype—are well established. However, recent studies have demonstrated that senescence is a heterogeneous process, with molecular markers varying according to the stressors that induce it. This review focuses on the functional aspects of MERCs in hepatic senescence and their impact on liver diseases, and explores the potential of targeting MERCs to address hepatocytic senescence.

## Inter-organelle communication through membrane contact sites

Compartmentalization in the form of organelles is one of the evolutionary advantages of eukaryotic cells, enhancing the efficiency of otherwise incompatible subcellular processes. There are approximately 20 types of major and minor organelles in a typical eukaryotic cell. Each organelle has its specific metabolic composition, pH, redox state, ionic composition, shape, and structural organization [1]. A typical organelle has an aqueous core that provides an optimal microenvironment for organelle-specific reactions and is surrounded by a phospholipid mono- or bilayer that separates it from the cytosol [2].

When the cell has to respond to a metabolic cue, organelles work in tight coordination to complete the task. For example, lipid metabolism involves the ER for lipid synthesis, lipid droplets (formed from the ER) for storage and transport, mitochondria and peroxisomes for β-oxidation, and lysosomes for lipid hydrolysis and recycling [3]. Thus, despite having individual identities, no organelle is an island and functions in isolation. Inter-organelle communication within a cell may occur via multiple modes such as diffusion, membrane-bound signaling pathways, vesicle trafficking, gap junctions, and membrane contact sites (MCSs). This review focuses on MCSs for inter-organelle communication.

**Fig. 1:**
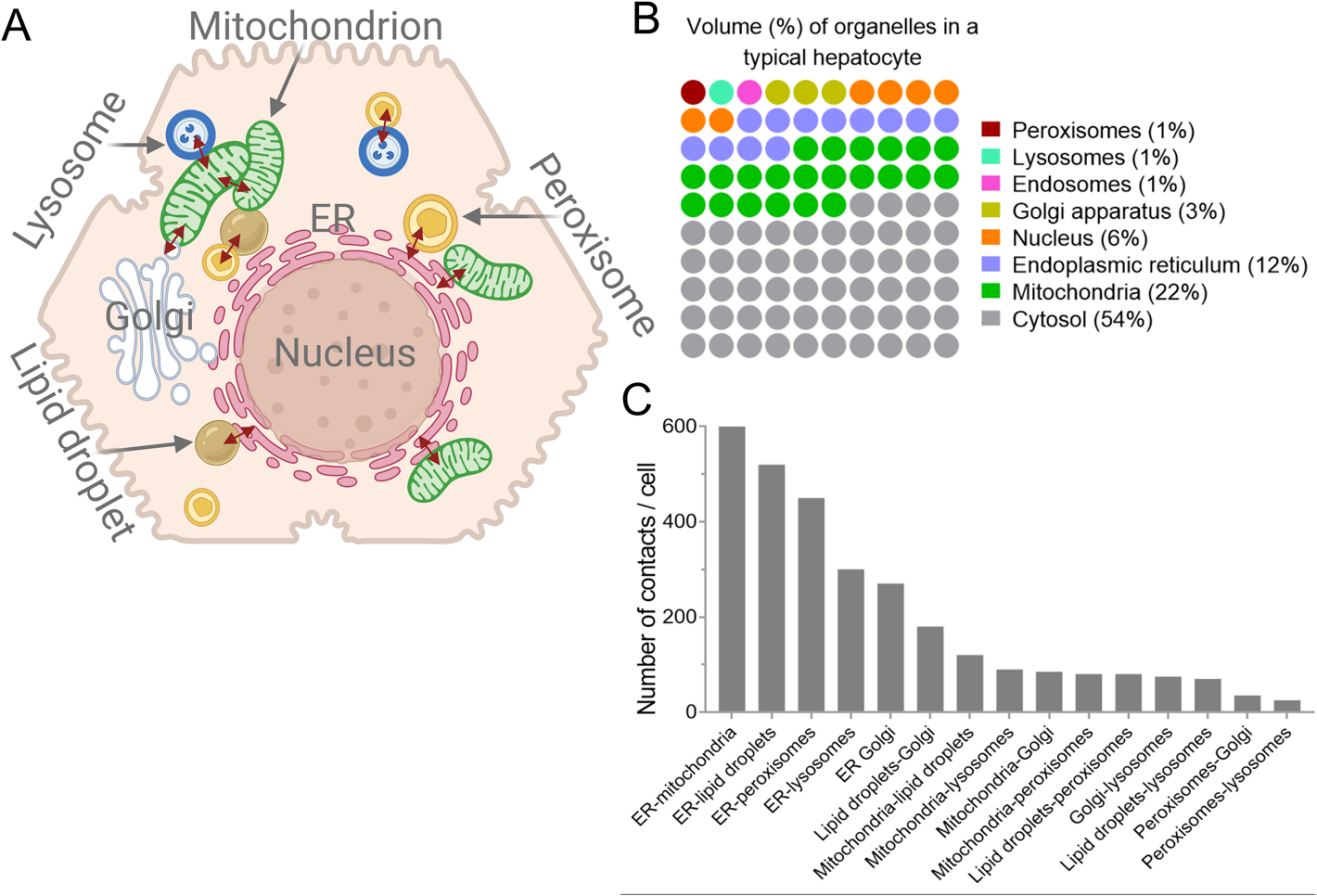
Inter-organelle communication via membrane contacts sites: **A** A schematic illustration depicting key inter-organelle tethering within a hepatocyte, with the bidirectional red arrow indicating inter-organellar interactions. **B** The proportion of cellular volume occupied by each organelle in a typical hepatocyte as described by Alberts et al. [181]. Each circle represents 1%, and the colors are assigned arbitrarily to improve visualization. **C** The number of contacts among various organelles per cell (fibroblast), as reported by Valm et al. [6]. Created with BioRender.com

MCSs are temporary molecular bridges that range from a few seconds to a few minutes, providing an efficient mode of communication between membrane-bound organelles (Fig. 1a) [4]. Mostly proteins and sometimes lipids are the structural components of these tethers, bringing organelles into proximity. The proteins involved in these tethers are categorized into four classes [5]:

1. Structural proteins: These form the skeleton of the contact sites and include tethers (e.g., ESyt1/2/3, ORP5/8, LAMs, VAPs, and Num1) that hold the two organelles together, and pillars/spacers (e.g., *E*-Syts) that inhibit the fusion of two membranes and keep them at a defined distance.
2. Functional proteins: These perform the exchange of metabolites at the contact site, including ion channels and pumps, metabolite channels/transporters, and lipid transfer proteins.
3. Sorter/recruitment proteins: These define the molecular niche of the contact site by recruiting or repelling proteins/lipids (Rab32 and PACS-2).
4. Regulator proteins: These regulate the function of the active proteins at the contact site, usually by post-translational modifications such as phosphorylation.

It is noteworthy that one protein may perform more than one function [5]. In terms of surface area, the ER is the largest organelle in a typical eukaryotic cell. It is a complex network of membrane-bound tubules and sacs that extend throughout the cytoplasm, occupying approximately 12% of the cell’s volume. Consequently, it is the most common partner in forming MCSs, contributing to approximately 72% of all the MCSs in a cell [6] (Fig. 1b). In the context of liver function, hepatocytes rely heavily on these ER-mediated MCSs to coordinate lipid metabolism, detoxification processes, calcium (Ca^2+^) signaling, protein trafficking, and ROS signaling.

## Mitochondria–ER contact sites (MERCs)

ER–mitochondria interactions were the first identified inter-organelle tethers and are generally referred to as mitochondria–ER contacts (MERCs). Vance was the first to isolate and characterize MERC fractions in rat liver and termed them “fraction X” [7]. MERCs are usually in the range of 10–80 nm and are involved in lipid transfer, Ca^2+^ and protein homeostasis, maintaining membrane dynamics, cell fate execution, and apoptosis [8, 9]. ER and mitochondria, which occupy up to 45% of cell volume, are involved not only in bioenergetics and macromolecular synthesis but also in controlling cell fate signaling. Therefore, the number of contact sites between ER and mitochondria is also highest in eukaryotic cells, approximately 600 sites per cell, covering up to 5% of the mitochondrial surface area (Fig. 1c) [6, 10]. MERCs are involved in lipid transfer, maintaining membrane dynamics, cell fate execution, cell signaling, and mitochondrial homeostasis. This makes MERCs a key intracellular mechanism to sustain hepatocyte functions in homeostasis and to adapt to injury in conditions of liver diseases.

Apart from their unique structure and architecture, MERCs have a characteristic set of proteins from each partner. The major mitochondrial protein partners forming MERCs are mitofusin 2 (Mfn2) [11], voltage-dependent anion-selective channel protein (VDAC) [12], mitochondrial fission 1 (FIS1) [13], and protein tyrosine phosphatase interacting protein 51 (PTPIP51) [14], and ER proteins include inositol 1,4,5-triphosphate receptor (IP3R) [15], oxysterol-binding protein-related protein (ORP) [15], and B-cell receptor-associated protein 31 (BAP31) [16]. There are also others proteins, such as, glucose-regulated protein 75 (GRP75), which is predominantly localized in the mitochondrial matrix, although a small subpopulation has also been observed in the cytoplasm and other compartments, including MERCs [17, 18, 35], and DJ-1 [19] acting as a connecting link between VDAC and IP3R, assisting in MERCs formation.

## Molecular exchanges via MERCs

### Ca^2+^ transfer and MERCs

**Fig. 2:**
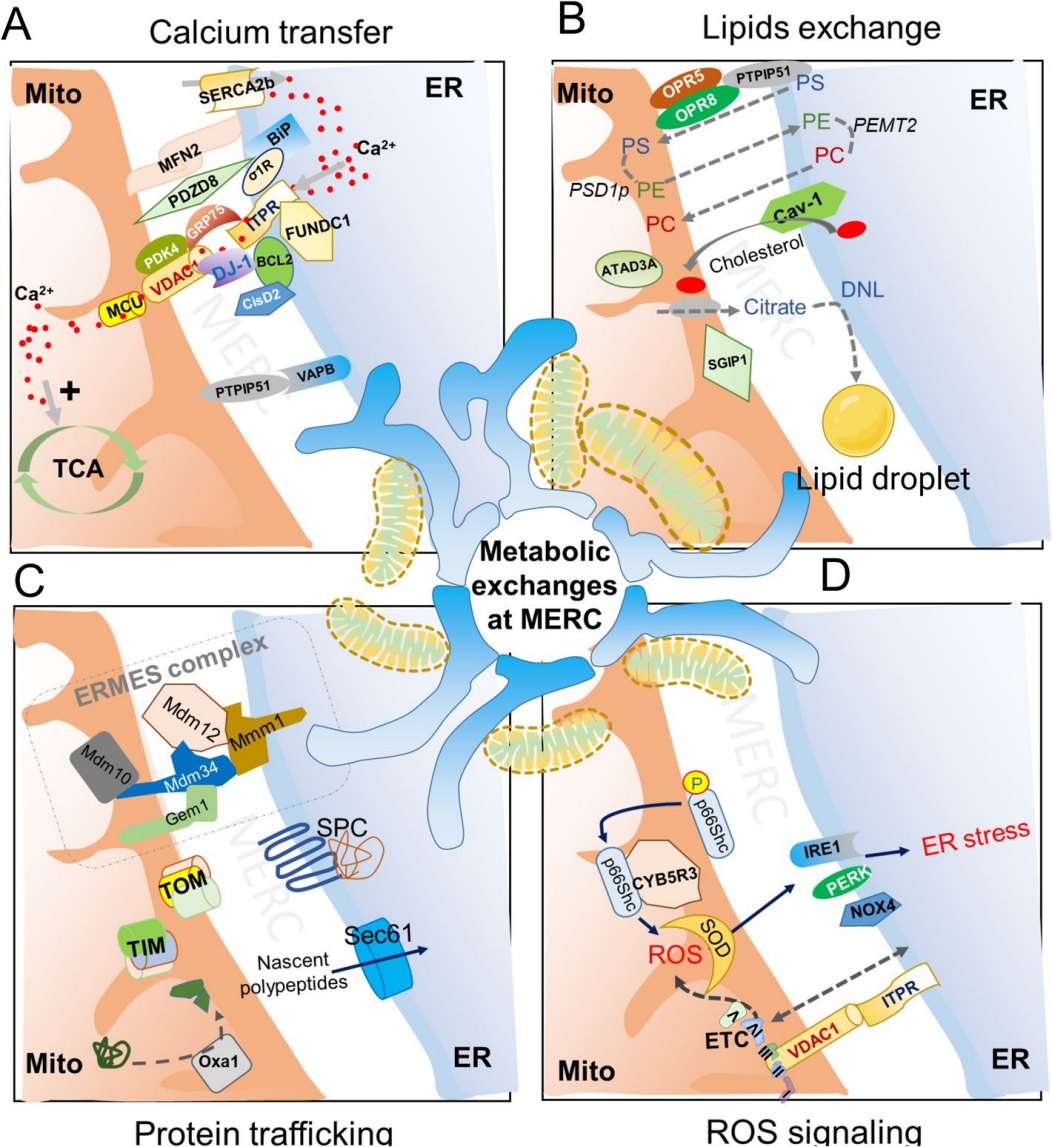
Functional roles of mitochondria–endoplasmic reticulum contact sites (MERCs) in metabolic exchange and signaling. MERCs act as critical hubs for inter-organelle communication, coordinating multiple cellular processes: **A** Calcium transfer: MERCs facilitate efficient Ca^2+^ shuttling from the ER to mitochondria through protein complexes involving IP3R–GRP75–VDAC1, MCU, and associated regulatory proteins (e.g., MFN2, PDZD8, DJ-1, and BCL2). This Ca^2⁺^ transfer supports mitochondrial metabolism, including the tricarboxylic acid (TCA) cycle, but excessive Ca^2+^ can promote mitochondrial stress. **B** Lipid exchange: MERCs mediate the transfer of phospholipids (phosphatidylserine [PS], phosphatidylethanolamine [PE], phosphatidylcholine [PC]) via enzymes such as PSD1p and PEMT2, as well as cholesterol and citrate transport involving proteins such as ATAD3A, caveolin-1 (Cav-1), and SGIP1. These exchanges contribute to lipid droplet formation and de novo lipogenesis (DNL). **C** Protein trafficking: MERCs participate in mitochondrial protein import through ER-associated translocation machinery (e.g., Sec61 complex and SPC) and mitochondrial import systems (TOM, TIM, and Oxa1), as well as ERMES complex proteins (Mdm10, Mdm12, Mdm34, and Gem1) that coordinate ER–mitochondria tethering. **D** ROS signaling: MERCs are sites of reactive oxygen species (ROS) generation and signaling, linking mitochondrial electron transport chain (ETC) activity to ER stress pathways via components such as CYP450 enzymes (CYPB5R3 and CYPB5R) and NOX4. ROS accumulation activates ER stress sensors (IRE1 and PERK), influencing cell fate and stress responses. Created with BioRender.com

Ca^2+^ and phosphates are two majors bivalent signaling ions in cells. Phosphate is part of structural units in nucleic acids and membranes, cycles among nucleotides (NAD–NADP, NADH–NADPH, AMP–ADP–ATP, and GMP–GDP–GTP), and affects protein structure and function by phosphorylation. In contrast, Ca^2+^ acts as a secondary messenger regulating enzymatic activity, ion channel function, and cytoskeleton movement. However, when Ca^2+^ and phosphate interact, they precipitate into Ca^2+^ phosphate, making both ions unusable in molecular signaling and inhibiting mitochondrial energy metabolism [20]. Eukaryotic cells strategically compartmentalize Ca^2+^ in the smooth ER (sER) and release it as needed. In the ER, most proteins directly or indirectly regulate Ca^2+^ homeostasis. Sigma-1 receptor (Sig-1R) is an ER protein that acts as a molecular chaperone and modulator of ER stress and Ca^2+^ signaling. Sigma-1 receptor (Sig-1R) is an ER protein that acts as a molecular chaperone and modulator of ER stress and Ca^2+^ signaling [21, 22]. Its role at MERCs involves regulating Ca^2+^ release from the ER to mitochondria, thereby influencing mitochondrial function and cellular stress responses (Fig. 2a). In its inactive state, Sig-1R is attached to the chaperone GRP78 within the ER [23]. However, during ER stress, such as Ca^2+^ depletion, this association breaks apart, and Sig-1R interacts with IP3R, stabilizing it and promoting Ca^2+^ influx [24]. Ca^2+^ entry into mitochondria is crucial for energy production because numerous mitochondrial enzymes involved in glycolysis and the tricarboxylic acid cycle require Ca^2+^ activation [25, 26]. Ca^2+^ enters the mitochondria through the mitochondrial Ca^2+^ uniporter complex (MCUcx), composed of five subunits, two of which (MCU and MCU-regulating EMRE) span the inner mitochondrial membrane, while three Ca^2+^-regulatory subunits (MICU1, MICU2, and MICU3) reside in the intermembrane space [27–29]. Ca^2+^ first crosses the OMM via the nonselective channel VDAC and reaches the IMS; from there, it reaches the matrix through the highly selective MCUcx [30]. It is noteworthy that MCU and EMRE are the first selective entry gates for mitochondrial Ca^2+^. A higher level of Ca^2+^ (10–20 µM) is necessary at MCUcx for its activation [31]. This elevated Ca^2+^ level is facilitated by the arrangement of MERCs, where Ca^2+^ release channels (IP3R and RyR) project from the ER surface, allowing proximity to the Ca^2+^ release site to the MCUcx via VDAC [32].

Several associated proteins assist the Ca^2+^ transfer from the ER to mitochondria [33]. For example, silencing GRP75 mitigates mitochondrial Ca^2+^ uptake owing to the disruption of IP3R–VDAC functional interaction [34, 35]. Disruption of another IP3R interactive protein, FUN14 domain containing 1 (FUNDC1), reduces Ca^2+^ levels in mitochondria [36]. Similarly, PDZ domain-containing protein 8 (PDZD8) regulates the levels of influx of Ca^2+^ into mitochondria, with its knockdown in neurons disrupting MERCs and decreasing mitochondrial Ca^2+^ uptake [37].

### Lipid transfer at MERCs

MERCs are active sites for lipid synthesis, and bidirectional transfer of lipids and associated metabolites occurs between the ER and mitochondria [38]. Lipid transfer ensures the supply of essential lipids required for membrane biogenesis and function, including the synthesis of mitochondrial-specific lipids such as cardiolipin [39].

For de novo lipogenesis, mitochondria export citrate, converted to acetyl-CoA by ATP citrate lyase (ACLY), the enzyme located at MERCs. However, no reports indicate that citrate transporters are enriched at MERCs. The de novo lipogenesis cascade in the cytosol continues with the formation of malonyl-CoA, palmitate, and triglycerides, then packaged into lipid droplets at the ER surface [40]. Conversely, mitochondria import phospholipids to form and maintain their membrane structure. Phospholipids shuttle between the ER and mitochondria, with PS formed at MERCs from either PC (by PSS1) or PE (by PSS2) and imported to mitochondria via ORP5/ORP8-PTPIP51 interaction [41, 42]. The imported PS is decarboxylated into phosphatidylethanolamine (PE) by PtdSer decarboxylase 1 (Psd1p) in the inner mitochondrial membrane, and the PE is transferred from mitochondria to the ER [43] (Fig. 2b). PE and cardiolipin maintain the tubular morphology of mitochondria and stabilize and activate ETC complexes [44]. Synj2bp Synaptojanin-2-binding protein (Synj2bp, also known as SGIP1) contributes to regulate the hepatic lipid flux by increasing MERCs in liver [45]. Synj2bp promotes the formation of MERCs by interacting with proteins involved in ER-mitochondria tethering, thereby enhancing lipid transfer and mitochondrial function in hepatocytes [45, 46]. This is particularly relevant in the context of MASLD, where increased lipid accumulation in the liver is a key pathological feature [47]. VAMP-associated protein B (VAPB) on the ER membrane and protein tyrosine phosphatase-interacting protein 51 (PTPIP51) on the OMM form a tethering complex at MERCs, stabilizing the physical interaction between ER and mitochondria and promoting lipid transfer [48]. ORPs are lipid-transfer proteins that shuttle specific phospholipids such as phosphatidylserine and phosphatidylinositol between the ER and other membranes, including mitochondria [49, 50]. ATPase associated with diverse cellular activities 3 A (ATAD3A), an ATPase associated with various cellular activities, is localized on the inner mitochondrial membrane and interacts with the outer mitochondrial membrane, maintaining mitochondrial structure and coordinating lipid transfer across mitochondrial membranes [51, 52]. Recent research has shown that splice isoforms of Mfn2 collaboratively regulate MERCS, Ca^2+^ transfer, lipid transport from the ER to mitochondria, and ER dynamics [53]. Mfn2 deficiency significantly disrupts phospholipid metabolism, acting upstream of the UPR, by impairing PS transfer from the ER to mitochondria and decreasing PS synthesis through reduced expression of PSS1 and PSS2. Additionally, it maintains phospholipid homeostasis, as it binds PS in vitro and promotes its partitioning into rigid membrane domains—activities that are specific to PS and do not occur with other phospholipids such as PE or PC [54].

### Protein trafficking at MERCs

Protein trafficking at MERCs involves importing proteins synthesized in the cytosol into mitochondria and exporting mitochondrial proteins back to the ER or other cellular compartments. This bidirectional trafficking is crucial for maintaining mitochondrial function and overall cellular homeostasis.

Mitochondrial oxidase assembly protein 1 (Oxa1) is a protein in the inner mitochondrial membrane that aids in the insertion of mitochondrially encoded proteins into the inner mitochondrial membrane (IMM), supporting the proper assembly of protein complexes within the IMM and working with imported OXPHOS components [55]. While Oxa1 is primarily involved in mitochondrial protein insertion and assembly, its function is essential for maintaining mitochondrial respiratory capacity and overall mitochondrial homeostasis [56]. Translocases of the outer mitochondrial membrane (TOM) complexes are the main entry gate for importing nuclear-encoded mitochondrial proteins [57]. Essential TOM complex subunits (TOM40, TOM20, and TOM70) recognize and translocate precursor proteins from the cytosol into the intermembrane space of mitochondria, supporting subsequent sorting and folding [58]. Translocases of the inner mitochondrial membrane (TIM) complexes (TIM23 and TIM22) facilitate importing proteins into the inner mitochondrial membrane (IMM) and the matrix, working in concert with TOM complexes to ensure proper protein insertion and folding [58].

Mitochondrial import machinery (MIM) complex components, such as MIM1, assist in inserting newly synthesized mitochondrial outer membrane proteins, ensuring correct localization of proteins integral to the OMM’s structure and function [59]. The signal peptidase complex (SPC) in the ER cleaves signal peptides from precursor proteins once targeted to the ER membrane, processing precursor proteins destined for mitochondria [60]. The Sec61 translocon in the ER membrane facilitates translocating nascent polypeptides into the ER lumen or membrane insertion, supporting mitochondrial precursor protein processing and sorting [61] (Fig. 2c). Mitochondrial Rho GTPase (Miro) proteins on the mitochondrial surface regulate mitochondrial motility and positioning, facilitating the capture of cytosolic precursor proteins for import [62]. Chaperone proteins (Hsp70 and Hsp60) assist in proper folding and assembly of mitochondrial proteins after import, preventing aggregation and ensuring functional integrity [63].

MERCs proteins create a physical bridge between the ER and mitochondria, facilitating lipid and protein transfer. Oxa1 in the IMM aids in inserting mitochondrially encoded proteins, supporting proper assembly of protein complexes within the IMM and working with imported OXPHOS components [64, 65].

### ROS signaling at MERCs

Reactive oxygen species (ROS) are byproducts of cellular metabolic processes, primarily generated in mitochondria during oxidative phosphorylation. While excessive ROS can cause oxidative damage, controlled ROS production and signaling are vital for various cellular processes, including energy metabolism, apoptosis, and immune responses [66, 67]. MERCs facilitate ROS signaling by maintaining close proximity between the ER and mitochondria, enabling efficient communication and metabolic signal exchange [68, 69].

The ETC, located in the inner mitochondrial membrane, generates ATP through oxidative phosphorylation. Electron leakage during transport can form superoxide (O_2_^−^) at complexes I and III, acting as a primary ROS signal [70]. This superoxide rapidly converts to hydrogen peroxide (H_2_O_2_), a more stable ROS species that participates in redox signaling [71].

Nicotinamide adenine dinucleotide phosphate oxidase (NOX) enzymes on the ER membrane generate ROS by transferring electrons from NADPH to oxygen, forming superoxide, contributing to localized ROS production, and influencing redox signaling pathways [72]. Their activation at MERCs can modulate Ca^2+^ signaling and mitochondrial function, impacting cellular senescence and liver disease progression. VDAC on the OMM serves as a gateway for ions and small molecules, including ROS, between the cytosol and mitochondria, regulating ROS release from mitochondria into the cytosol and ER [73]. Superoxide dismutase (SOD) enzymes convert superoxide radicals to hydrogen peroxide, mitigating oxidative stress and ensuring superoxide generated at MERCs is converted to H_2_O_2_. By converting superoxide to hydrogen peroxide, SOD ensures that ROS generated at MERCs are converted into a more stable and diffusible form, which can act as a signaling molecule or be further detoxified [74].

Also, 66Shc is a redox-regulating protein that localizes variably within the cytosol, ER, mitochondria, and MERCs with its distribution influenced by cellular stress and experimental approaches. Its accumulation in MAM suggests a role in regulating mitochondrial function, oxidative stress, and apoptosis, especially through interactions at ER–mitochondria contact sites critical for Ca^2+^ signaling and lipid metabolism [75]. Notably, p66Shc translocates to MAM under oxidative stress conditions, where it may influence ROS production by interacting with mitochondrial proteins such as NADH-cytochrome b5 reductase 3 (CYB5R3) [75] (Fig. 2d).

## MERCs in mitochondrial homeostasis

### MERCs, mitochondrial dynamics, and senescence

Mitochondria are dynamic organelles continuously undergoing processes such as fission and fusion, which are regulated by a host of proteins. These processes are crucial for maintaining mitochondrial integrity, enabling quality control, and ensuring appropriate cellular distribution [76]. In healthy cells, a balance between mitochondrial fusion and fission is necessary for proper mitochondrial function. Mitochondrial fission and fusion are spatially coordinated at MERCs to regulate mitochondrial morphology. Mitofusins, key components of the mitochondrial fusion machinery, accumulate at MERCs, facilitating fusion events. These MERCs act as dynamic hotspots capable of undergoing both fission and fusion, enabling rapid responses to metabolic cues. MERCs define the boundaries between polarized and depolarized segments of mitochondria, suggesting a role in mitochondrial quality control [77].

MERCs are vital for coordinating these dynamics. During fission, dynamin-related protein 1 (Drp1) is recruited from the cytosol to the mitochondrial surface by receptor proteins such as Fis1, MFF, MiD49, and MiD51 located on the outer mitochondrial membrane. The ER wraps around the mitochondrion at the fission site, creating a constriction point [78]. Drp1 assembles into a ring-like structure around the mitochondrion at these constriction sites [79]. GTP hydrolysis by Drp1 provides the energy to tighten this ring, further constricting and ultimately severing the mitochondrion into two separate organelles, resulting in mitochondrial biogenesis [80].

In contrast, during fusion, MERCs help coordinate the activities of mitofusins and OPA1 by managing the lipid environment and supplying necessary resources for membrane merging [81]. When two mitochondria come into proximity, Mfn1 and Mfn2 proteins on adjacent mitochondria interact in a GTP-dependent manner, tethering the outer membranes together and facilitating their merging [82]. Following outer membrane fusion, OPA1, which regulates inner membrane fusion, facilitates the merging of the inner mitochondrial membranes to ensure the complete fusion of the two mitochondria [83, 84].

In senescent cells, there is often a marked reduction in the expression of key fusion proteins such as Mfn1, Mfn2, and Optic Atrophy 1 (OPA1) [85, 86]. This reduction impairs the mitochondria’s ability to undergo fusion, leading to fragmentation [87]. MFN1 is a substrate of the ubiquitin ligase MARCH5; in cells lacking MARCH5, MFN1 accumulates, leading to hyperfused mitochondria and features associated with senescence [88]. Conversely, suppression of MFN1 has been shown to extend the replicative lifespan [87]. MFN2, on the other hand, when knocked down, promotes proliferation in both B cell lymphoma lines and mouse embryonic fibroblasts [89].

The enzymatic activities and structural integrity of fusion proteins may also be compromised, further hindering the fusion process [90, 91]. Senescent cells typically exhibit upregulation of fission proteins like Drp1, which promotes mitochondrial fragmentation. Increased activity of Drp1 and its receptors (MFF, Fis1, MiD49, and MiD51) leads to excessive mitochondrial fission, resulting in fragmented mitochondrial networks [92, 93]. These fragmented mitochondria often exhibit impaired bioenergetic functions, producing ATP less efficiently. Additionally, fragmented mitochondria are associated with increased ROS production, further damaging mitochondrial DNA (mtDNA), proteins, and lipids, exacerbating cellular aging [94]. Elevated ROS levels contribute to oxidative stress, promoting cellular damage and further accelerating the onset of senescence [95]. Altered mitochondrial dynamics contribute to the SASP, which involves the secretion of pro-inflammatory cytokines, chemokines, and proteases, exacerbating tissue degeneration and aging [96–98]. In the tethering complex ITPR–GRP75–VDAC, GRP75 overexpression has been reported to extend replicative lifespan by downregulating RAS signaling and decreasing ERK2 phosphorylation [89]. Silencing any of the ITPR isoforms (ITPR1, ITPR2, or ITPR3) in human mammary epithelial cells prevents oncogene-induced senescence, while in normal human fibroblasts, it delays replicative senescence [99]. ITPR2 acts both as a Ca^2+^ channel and a structural tether promoting MERC formation. Loss of Itpr2 reduced senescence levels both in vitro and in vivo, leading to delayed age-related liver decline, including reduced steatosis and fibrosis, and improved metabolic responses. Mechanistically, ITPR2 promoted senescence partly through its role in maintaining MERCs, leading to mitochondrial Ca^2+^ overload and ROS production, which induce p53-dependent senescence. Additionally, increased MERC formation enhanced pro-inflammatory pathways involving NF-κB and inflammasome activation, promoting a SASP [100].

### MERCs and autophagy

Autophagy is a crucial process for maintaining cellular homeostasis by degrading and recycling cellular components. The exact origin of autophagosome membranes has been a subject of study, with various theories suggesting contributions from multiple organelles, including the ER, mitochondria, and plasma membrane. Recent research highlights the significant role of MERCs in autophagosome formation in mammalian cells.

ATG14, a key protein in autophagy, is a subunit of the autophagy-specific PI(3)K complex essential for autophagosome formation. During starvation-induced autophagy, ATG14 localizes to the ER and assembles at specific membrane points [101]. STX17, an ER-resident SNARE protein, plays a pivotal role in recruiting ATG14 to the MERCs, crucial for autophagosome formation [102]. Further investigations into STX17 demonstrated its essential function in autophagosome maturation, with its knockdown leading to the accumulation of isolation membranes deficient in autophagosome completion [103].

Another protein, Miga, interacts with Uvrag and Atg14 to regulate PI3P production and stabilize Syx17, linking MERCs to autophagy. Miga’s stabilization of Syx17 through Atg14 is crucial for autophagosome-lysosome fusion, with ERMCSs serving as platforms for lipid and protein transfer necessary for autophagy progression. These interactions influence hepatocyte lipid accumulation and senescence by affecting autophagic clearance and organelle homeostasis, with Mfn2 modulation similarly impacting lipid metabolism and cell aging through MERC dynamics [104–106].

Phosphofurin acidic cluster sorting protein-2 (PACS-2) and MFN2 also facilitate ER-mitochondria contacts and autophagosome formation [107]. Live-cell imaging studies reveal that ATG5, a marker for isolation membranes that develop into autophagosomes, localizes predominantly at the ER-mitochondria contact site during autophagy initiation [102]. Disruption of this localization hinders autophagic flux and inhibits the formation of functional autophagosomes.

Moreover, VAP proteins on the ER membrane interact with proteins like PTPIP51 on the mitochondrial membrane, tethering the organelles together and facilitating lipid exchange, essential for autophagic processes [108]. Mitofusins (MFN1 and MFN2), involved in mitochondrial fusion, establish and maintain mitochondria-ER contacts critical for autophagosome formation [109]. IP3R on the ER membrane regulates Ca^2+^ release into the cytosol, influencing autophagy and apoptosis at MERCs [110]. DRP1, a regulator of mitochondrial fission, impacts mitochondria-ER contact sites and autophagy by segregating damaged mitochondria for selective autophagy [111]. Beclin1 plays a crucial role in autophagy and mitophagy by interacting with proteins like Uvrag and Atg14 to promote PI3P production at MERCs ensuring autophagosome formation near damaged mitochondria for efficient degradation. Its localization to MERCs during mitophagy depends on Ulk1-mediated phosphorylation at Ser15, which facilitates autophagosome initiation specifically in response to stress, whereas Beclin2 does not share this function. Under basal conditions, both Beclin1 and Beclin2 contribute to autophagosome formation, but stress activates a Beclin1/2-independent pathway that can bypass their requirement, preserving autophagic activity. Mechanistically, Beclin1’s regulation at MERCs links autophagy directly to lipid metabolism, as MERCs serve as platforms for lipid transfer essential for autophagosome biogenesis, with phosphorylation at Ser15 modulating its recruitment and function during stress-induced autophagy [112, 113].

**Fig. 3:**
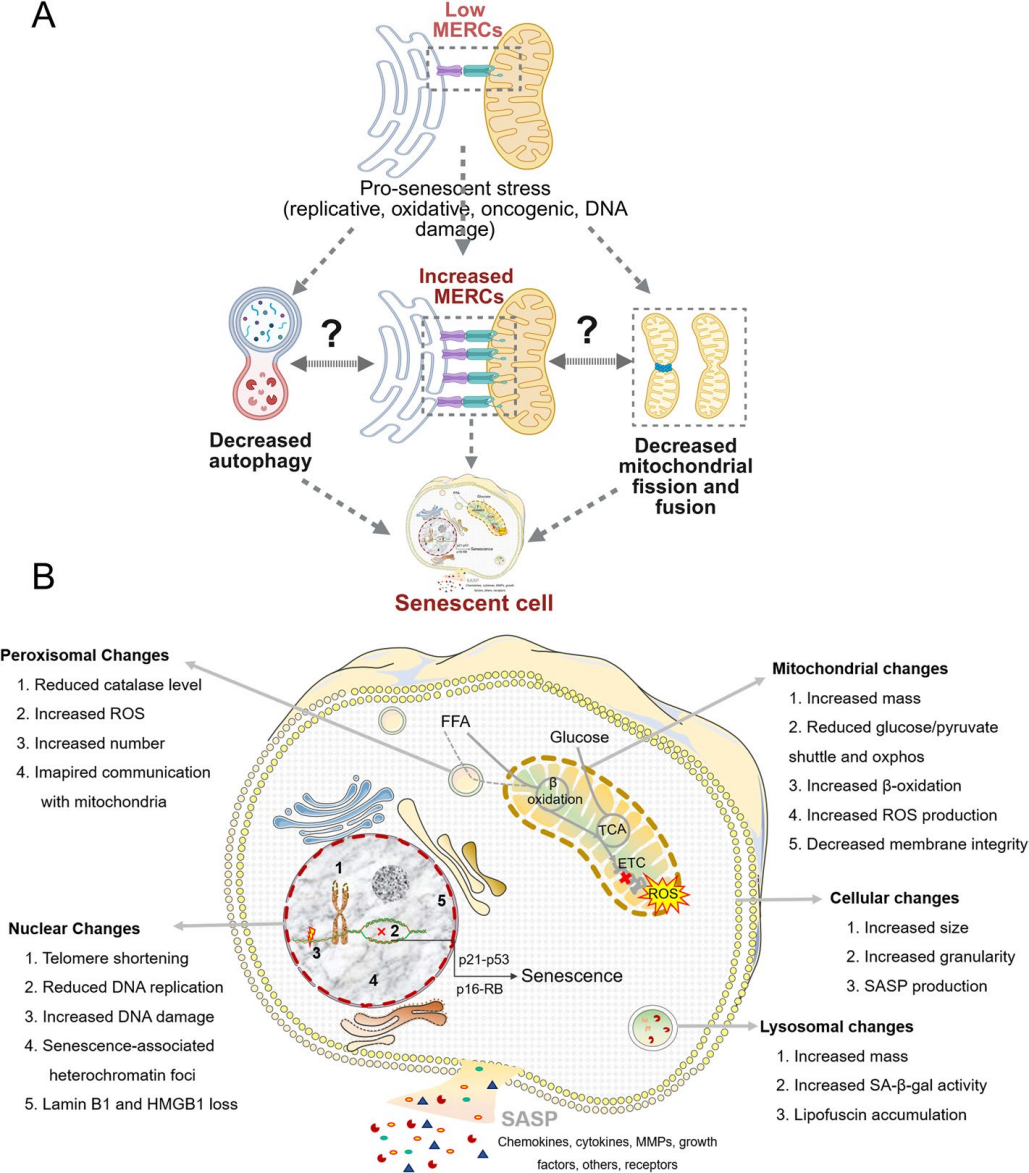
MERC remodeling, and cellular senescence. **A** Pro-senescent stressors, such as oxidative stress, lipid overload, inflammation, and viral infection, trigger initial reductions in mitochondria–endoplasmic reticulum contact sites (MERCs), followed by a compensatory increase during the establishment of senescence. Increased MERCs disrupt inter-organelle communication, contributing to decreased mitochondrial fission and fusion, impaired autophagy, and sustained mitochondrial dysfunction. These MERC-driven processes contribute to hepatocellular senescence and the development of the senescence-associated secretory phenotype (SASP), which further propagates liver injury and fibrosis. Dashed arrows indicate proposed mechanistic links; question marks denote relationships requiring further clarification. **B** Intracellular molecular characteristics of a typical senescent cell: Senescence involves several key changes. Morphologically, senescent cells exhibit increased size and granularity. Within the nucleus, there is a loss of nuclear membrane integrity, telomere shortening, DNA damage, and the formation of senescence-associated heterochromatin foci. In peroxisomes, changes include reduced catalase activity, increased reactive oxygen species (ROS), an increase in number, and impaired communication with mitochondria. Mitochondrial changes include increased mass, reduced bioenergetic efficiency, heightened ROS production, and decreased membrane integrity. In lysosomes, there is an increase in mass, elevated SA-β-galactosidase activity, and the accumulation of lipofuscin. Created with BioRender.com

The relationship between MERCs and cellular senescence is complex, with studies reporting seemingly contradictory findings regarding their role (Fig. 3a).

Research has identified both direct and inverse correlations between the number of MERCs and the induction of senescence. This suggests a need for further examination of specific types of senescence to better understand these dynamics. Our recent study demonstrated that, in hepatocytes, different stressors activate distinct markers of senescence [114], indicating that senescence is not a uniform process but a highly heterogeneous one. Similarly, it is important to investigate the variety of MERCs in the context of their specific protein partners and interactions, which may shed light on their diverse roles in cellular senescence. Given that senescence is an underlying factor in many chronic liver diseases, we next explore the connections between MERCs, senescence, and relevant liver diseases in more detail.

### Pro-senescent role of MERCs

Multiple pro-senescence stresses, including replicative aging, oxidative stress, oncogenic activation, and DNA damage, have been shown to modify the protein composition of MERCs. For instance, persistent DNA damage, such as that occurring at telomeres during replicative senescence or after X-ray exposure, upregulates the expression of BAP31, a key MERC tether, at the mRNA level [115]. Similarly, ITPR2, the most efficient channel for Ca^2+^ transfer from the ER to mitochondria, is transcriptionally upregulated by various senescence-inducing conditions, including high-fat diet exposure [116], oncogenic stress, and oxidative insults [99]. Beyond transcriptional regulation, stress-induced activation or re-localization of MERC-associated proteins, such as p66Shc, further underscores the responsiveness of MERCs to cellular damage and stress.

Artificial enhancement of MERCs, by enforcing close ER–mitochondrial proximity using synthetic linkers, has been shown to trigger premature senescence in normal human fibroblasts [117]. This response is accompanied by increased mitochondrial Ca^2+^ uptake and elevated ROS production, which could be reversed by antioxidant treatment. Mechanistically, this ROS-driven senescence requires p53 activation and involves a NF-κB-dependent senescence-associated secretory phenotype (SASP) [100]. These findings suggest that forced ER–mitochondrial coupling initiates a Ca^2+^–ROS–p53 axis that drives senescence. In contrast, uncoupling MERCs, such as through the deletion of associated proteins like MFN2, Frataxin, or ORP5, has also been associated with senescence-like phenotypes [42, 118, 119]. However, whether these effects are MERC-dependent or arise from broader cellular dysfunction remains unclear.

Ca^2+^ transfer from ER to mitochondria through MERCs, emerges as a central regulator [120, 121]. Replicative senescence is marked by increased MERCs and mitochondrial Ca^2+^ overload [122], whereas reducing this Ca^2+^ flux, via knockdown of ITPR2 or MCU, attenuates senescence markers across multiple cell types, including fibroblasts and mammary epithelial cells [99].

MERCs also coordinate mitochondrial fission by serving as platforms where ER tubules wrap around mitochondria to define division sites. Inhibition of fission in normal cells has been shown to induce senescence, likely due to the accumulation of hyperfused, dysfunctional mitochondria and defective mitophagy [97, 123]. While it remains unproven whether MERC uncoupling alone causes senescence, it may contribute by disrupting fission and quality control mechanisms.

In another MERC partner, ER, chronic stress is a recognized contributor to senescence across various models [124–126]. ER stress can stem from prolonged unfolded protein response (UPR) activation or luminal Ca^2+^ depletion, mediated through PERK, ATF6, and IRE1 signaling. Intriguingly, MFN2 depletion, disrupting ER–mitochondria tethering, increases the UPR, highlighting a direct link between MERC dysfunction and ER stress-induced senescence [127, 128] (Fig. 3a).

## Hepatocellular senescence and MERCs in liver diseases

Traditionally, it has been assumed that a primary cell meets one of two fates: (1) apoptosis, a process in which cells differentiate themselves to death, or (2) cancer, an uncontrolled/unchecked cell proliferation. In both processes, the cell has to keep “cycling.” However, in 1961, Hayflick and Moorhead observed that serial cultivation of human diploid fibroblasts resulted in altered morphology of the cells and loss of proliferative capacity despite the appropriate nutrients and proliferative culture conditions and named this senescence [129]. Later on, senescence was studied extensively and described as a state of metabolically active irreversible cell cycle arrest. Therefore, a cell stays in a “zombie phase” at the crossroads of apoptosis and proliferation pathways for a long period. Senescence phenotype is a heterogeneous and dynamic and multistep process of continuously evolving senescent properties in a cell (Fig. 3b) [114, 130–132].

Senescent cells progressively accumulate during aging, thus it is considered one of the hallmarks of aging [133]. Typical characteristics of a senescent cell include cell cycle withdrawal, macromolecular (proteins, DNA, lipids) damage, secretion of chemokines and cytokines (SASP), increased lysosomal content, and accumulation of dysfunctional mitochondria; however, the sequence and combinations of these changes may vary depending on the cell type and stimulus [134].

Activation of the p53/p21WAF1/CIP1 and p16INK4A/pRB tumor suppressor pathways plays a central role in regulating senescence [135, 136]. Developmentally programmed senescence is mediated by p21 regulated by TGF-β/SMAD and PI3K/FOXO pathways, and generally independent of DNA damage and p53 [137].

Whether senescence is beneficial or harmful strongly depends on the biological context; For example, it is beneficial and helps pattern formation during embryogenesis, wound healing, and tumor suppression. However, it has detrimental effects during aging-related diseases, cell injury, and tissue regeneration. Therefore, cellular senescence acts as a double-edged sword and is thereby considered to be an example of evolutionary antagonistic pleiotropy [97, 130].

**Fig. 4:**
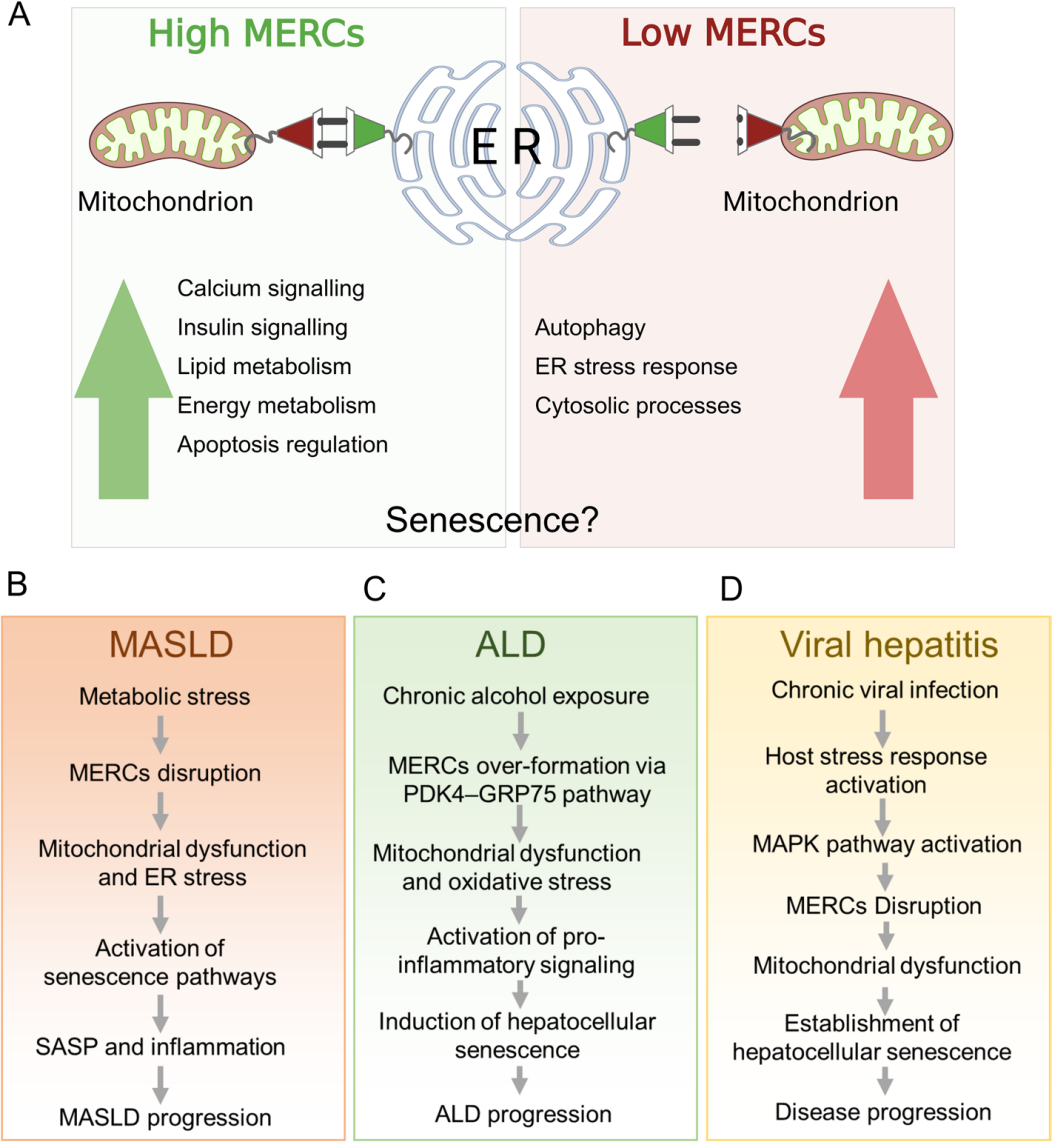
Impact of MERCs on cellular processes in liver diseases. **A** A high number of mitochondrial–ER contact sites (MERCs) is associated with enhanced Ca^2+^ signaling, insulin signaling, lipid metabolism, energy metabolism, and the regulation of apoptosis. Conversely, a low number of MERCs is linked to increased autophagy, an enhanced ER stress response, and various cytosolic processes. MERC-mediated mechanisms linking hepatocellular senescence to chronic liver disease progression in MASLD, ALD, and viral hepatitis. **B** In metabolic dysfunction-associated steatotic liver disease (MASLD), metabolic stress induces disruption of mitochondria–endoplasmic reticulum contact sites (MERCs), leading to mitochondrial dysfunction, ER stress, and activation of senescence pathways. The resulting senescence-associated secretory phenotype (SASP) drives inflammation and disease progression. **C** In alcohol-related liver disease (ALD), chronic alcohol exposure promotes excessive MERCs formation via the PDK4–GRP75 pathway, causing mitochondrial Ca^2+^ overload, oxidative stress, and activation of pro-inflammatory signaling, culminating in hepatocellular senescence and ALD progression. **D** In viral hepatitis, chronic HBV or HCV infection triggers host stress responses and MAPK pathway activation, disrupting MERCs and impairing mitochondrial function. These events facilitate the establishment of hepatocellular senescence and contribute to ongoing liver injury and disease progression. Created with BioRender.com

Although MERCs have been extensively studied in relation to senescence, their precise contributions to hepatic senescence are less well-defined. Table 1 compiles the available research in this area, which we will discuss in greater detail within the liver disease-specific subsections. Further research is needed to fully elucidate the role of MERCs in hepatic senescence (Fig. 4a).

**Table 1:** Key MERC proteins in senescence and liver disease

Protein	Role in MERCs	Role in senescence	Associated liver disease(s)	Refs.
IP3R	Mediates ER-to-mitochondria Ca^2+^ transfer; part of MERC tethering	Promotes mitochondrial Ca^2+^ overload; upregulated in senescence	MASLD, liver aging; liver fibrosis—via Ca^2+^ signaling pathways	[15, 100, 120, 138]
Mfn2	Maintains MERC structure; regulates lipid transfer and Ca^2+^ signaling	Reduces fusion; its deficiency leads to mitochondrial fragmentation and senescence	MASLD, liver aging	[11, 45, 54]
VDAC	Gatekeeper for ions and small molecules at OMM; involved in ROS signaling	Regulates ROS release; overactive in senescence	MASLD, alcohol-related liver diseases, viral hepatitis	[12, 68, 73]
GRP75 (mortalin)	Connects IP3R and VDAC, stabilizing ER-mitochondria tethering	Overexpression extends lifespan; involved in redox signaling	MASLD, liver aging	[17, 89, 100]
FIS1	Outer mitochondrial membrane protein involved in fission	Upregulated in senescence; promotes fragmentation	Liver aging, liver injury	[13, 86]
Drp1	Facilitates mitochondrial fission at MERCs	Increased activity leads to excessive fission and senescence	Liver aging, liver injury	[87, 92]
MIGA	Links MERCs to autophagy and lipid regulation	Stabilizes MERCs, promotes autophagy, influences lipid metabolism	Liver steatosis, MASLD	[104–106]
PTPIP51	Tethering at MERCs; involved in lipid transfer	Disruption linked to mitochondrial dysfunction and senescence	MASLD, liver injury	[14, 48]
SIG-1R (Sigma-1 receptor)	Chaperone modulating Ca^2+^ signaling at MERCs	Regulates ER stress and Ca^2+^ signaling; influences senescence	Liver diseases, stress response	[21, 22]
ERO1α	Located at MAM; involved in disulfide bond formation and ER stress	ER stress induction; linked to senescence and aging	MASLD, liver aging	[139, 140]

### MERCs impact in metabolic dysfunction-associated steatotic liver disease

Metabolic dysfunction-associated steatotic liver disease (MASLD) is characterized by fat accumulation in liver cells, insulin resistance, and metabolic syndrome components [141]. MERCs play pivotal roles in facilitating metabolic coordination and stress responses in both MASLD and cellular senescence [9, 142, 143]. Several proteins involved in these processes, such as Mfn2, IP3Rs, VDAC, NOX, SOD, GRP78, ERO1α, and UPR sensors, have dual roles in maintaining cellular homeostasis and responding to metabolic stress [144–146]. Mfn2 is essential for maintaining MERC structure and function, with its disruption leading to impaired Ca^2+^ signaling, mitochondrial dysfunction, and increased ER stress [11]. These effects have been mechanistically demonstrated to occur through MERCs, contributing to MASLD progression. Inositol 1,4,5-triphosphate receptors (IP3Rs) regulate Ca^2+^ release from the ER to mitochondria, modulating mitochondrial function and cellular stress responses [120, 147]. The pathogenic roles of IP3Rs in liver disease occur specifically via MERCs, influencing mitochondrial Ca^2+^ uptake and downstream signaling pathways [148]. The voltage-dependent anion channel (VDAC) facilitates the exchange of ions and metabolites between mitochondria and the cytoplasm, impacting mitochondrial function and cellular metabolism. The role of VDAC in liver disease occurs via MERCs by regulating the transport of metabolites and ions necessary for maintaining mitochondrial homeostasis and energy production [149–151]. NOX enzymes are key sources of ROS in the ER membrane, contributing to oxidative stress and redox signaling pathways. Their activation at MERCs can modulate Ca^2+^ signaling and mitochondrial function, impacting cellular senescence and liver disease progression. ROS at the MAM positively influences local mitochondrial Ca^2+^ flux [152, 153]. SOD enzymes are antioxidant enzymes that catalyze the dismutation of superoxide radicals into hydrogen peroxide and oxygen, thereby reducing oxidative stress. By converting superoxide to hydrogen peroxide, SOD ensures that ROS generated at MERCs are converted into a more stable and diffusible form, which can act as a signaling molecule or be further detoxified [74]. GRP78 is an ER chaperone that manages protein folding, with dysregulation exacerbating liver injury and inflammation [154]. The effects of GRP78 in liver disease occur through MERCs by regulating ER stress responses and influencing Ca^2+^ signaling between the ER and mitochondria [150]. ERO1α is primarily confined to the MAM contact sites within the endoplasmic reticulum. A decrease in ERO1α levels leads to disruptions in mitochondrial Ca^2+^ transfer and impairs mitochondrial respiratory capacity [139].

Dysfunction in these proteins can drive MASLD progression and accelerate cellular aging, linking metabolic diseases with a broader context of age-related cellular decline [140, 155]. Dysregulation can lead to lipid accumulation in hepatocytes.

Recently, Bassot et al. demonstrated that both silencing and overexpression of Grp75 or Mfn2 markedly affect hepatic lipid and cholesterol metabolism by disrupting the integrity of MERCs. Silencing these proteins increases triglyceride accumulation due to impaired mitochondrial fatty acid oxidation, while overexpression induces lipid build-up through ER stress, altered phospholipid synthesis, and defective ApoB100 lipoprotein secretion. ER stress appears to be a key mediator linking MERCs alterations to lipid accumulation, as its relief with 4-PBA prevents these metabolic disturbances [53].

SCP-2 manages the transfer of cholesterol and phospholipids, with its impairment disrupting lipid metabolism and leading to steatosis [156, 157]. MERCs also play roles in protein quality control, with disruptions leading to ER stress and subsequent activation of the unfolded protein response (UPR) [158]. GRP78 (BiP), an ER chaperone, manages protein folding, with dysregulation exacerbating liver injury and inflammation [154]. UPR sensors such as ATF6, IRE1, and PERK mediate ER stress responses, affecting lipid metabolism and inflammatory responses [159].

Emerging evidence suggests that alterations in MERC dynamics impact MASLD pathogenesis [54, 160–162]. Disruptions in MERCs lead to mitochondrial dysfunction, impaired lipid metabolism, de novo lipogenesis, and increased oxidative stress, all contributing to MASLD progression. The complex interplay between hepatocellular senescence, PPAR-alpha, NF-kB, JNK pathways, and MERCs highlights intricate cellular interactions in liver disease progression. Further research into these mechanisms may help in developing novel targeted therapeutic interventions for MASLD (Fig. 4b).

### Involvement of MERCs in alcohol-related liver diseases

Alcohol-related liver diseases (ALD) are characterized by hepatocellular damage due to excessive alcohol consumption. One of the key factors in ALD progression is hepatocellular senescence [163]. Several proteins and signaling pathways regulating senescence are involved in the pathogenesis of ALD, with significant potential links to MERCs. Chronic alcohol exposure influences MERC structure and function, exacerbating mitochondrial dysfunction, oxidative stress, and lipid accumulation in hepatocytes [164].

Alcohol promotes the formation of MERCs and MCC complexes via PDK4-mediated phosphorylation of GRP75, leading to enhanced Ca^2+^ transfer from ER to mitochondria, which causes mitochondrial Ca^2+^ overload and oxidative stress—key contributors to mitochondrial dysfunction in ALD. Elevated PDK4 expression amplifies MERCs formation and MCC complex assembly, exacerbating mitochondrial stress, lipid accumulation, and liver injury, while PDK4 deficiency prevents these effects by disrupting MERCs integrity [138]. Alcohol-induced mitochondrial dysfunction inhibits fatty acid oxidation, leading to intrahepatic lipid accumulation [165, 166].

Nuclear factor erythroid 2-related factor 2 (Nrf2) plays a crucial role in ALD. Nrf2 is a transcription factor orchestrating the antioxidant response, defending cells against oxidative stress. Chronic alcohol consumption disrupts Nrf2 signaling, increasing oxidative damage, and promoting hepatocellular senescence [167]. The Toll-like receptor 4 (TLR4) signaling pathway is another significant mechanism in ALD, with chronic alcohol exposure activating TLR4 and triggering inflammatory responses. TLR4-mediated pathways play pivotal roles in developing and progressing ALD and hepatocellular senescence [155, 168, 169]. The transforming growth factor-beta (TGF-β) signaling pathway is implicated in alcohol-induced liver fibrosis and senescence, promoting extracellular matrix production and inducing hepatic stellate cell activation [170].

Disruptions in MERC dynamics further exacerbate mitochondrial dysfunction and oxidative stress in ALD. Alcohol-induced alterations in MERC structure and function contribute to impaired lipid metabolism, disrupted Ca^2+^ homeostasis, and increased cellular stress responses, all associated with hepatocellular senescence in ALD [138]. Understanding the interplay between proteins and pathways such as Nrf2, TLR4, TGF-β, and MERCs may facilitate the search for novel targeted therapeutic interventions for ALD (Fig. 4c).

### Role of MERCs in viral hepatitis

Chronic viral hepatitis, caused by hepatitis B or C viruses (HBV, HCV), leads to significant liver damage and disease progression. Hepatocellular senescence contributes to these outcomes, with several proteins and signaling pathways involved [171, 172].

The retinoblastoma protein (Rb) is essential in viral hepatitis-induced senescence [173]. Rb regulates cell cycle progression, and its dysregulation in viral hepatitis leads to cell cycle arrest and senescence in infected hepatocytes. The p53 pathway, a critical tumor suppressor, responds to cellular stress and DNA damage, promoting cell cycle arrest and senescence. Activation of p53 in infected hepatocytes can induce senescence as a host defense mechanism against viral replication [174]. The mitogen-activated protein kinase (MAPK) signaling pathway, involved in regulating inflammation and cellular responses, modulates senescence-associated phenotypes in infected hepatocytes, contributing to liver damage and disease progression [175].

Alterations in MERC dynamics significantly impact mitochondrial function and cellular metabolism in viral hepatitis [176]. Disruptions in MERC structure and function can influence viral replication, inflammatory responses, and cellular stress, contributing to hepatocellular senescence and liver injury [177]. The complex interactions between proteins and pathways such as Rb, p53, MAPK, and MERCs underscore the intricate mechanisms underlying hepatocellular senescence in viral hepatitis (Fig. 4d).

## Senolytics as potential treatments targeting MERCs

Senolytics, a class of drugs that selectively induce the death of senescent cells, hold promise in treating age-related diseases by restoring cellular functions and homeostasis [178]. These drugs may impact MERCs, which are crucial for cellular communication and the transfer of lipids, Ca^2+^ ions, and other signaling molecules [143]. Senolytics may help restore the normal morphology and function of ER–mitochondria interfaces by removing dysfunctional senescent cells, potentially rejuvenating cellular interactions and improving overall cell function. By targeting and eliminating senescent cells, senolytics reduce the burden of ROS and oxidative damage, potentially enhancing ER and mitochondrial function [178, 179]. This reduction in oxidative stress may reverse some of the detrimental effects associated with cellular senescence. Efficient ER–mitochondria communication is vital for Ca^2+^ signaling. Clearing senescent cells with senolytics enhance the regulation of Ca^2+^ transfer between the ER and mitochondria, improving cellular signaling and function [100].

Recently, Puebla-Huerta et al. investigated therapy-induced senescence (TIS) and its impact on Ca^2+^ fluxes at MERCs, identifying new senolytic targets. The study induced TIS using doxorubicin and etoposide, observing increased MERCs contact surface but decreased ER–mitochondria Ca^2+^ flux. Mechanistically, TIS cells showed reduced expression of IP3R isoforms and impaired interaction between type 1 IP3R and VDAC1, hindering Ca^2+^ transfer. Inhibition of this ER–mitochondria Ca^2+^ flux demonstrated senolytic effects in vitro and in vivo using desmethyl XeB (dmXeB), an IP3R inhibitor, reducing senescent cell burden in aged p16-3MR transgenic mice. The findings highlight the critical role of ER-mitochondria Ca^2+^ flux for the survival of TIS cells, positioning it as a promising target for senolytic interventions [120].

Preclinical studies have shown promise for senolytics in treating liver fibrosis, reducing inflammation, and improving metabolic profiles in models of MASLD and alcohol-related liver disease (ALD) [100, 180]. For instance, dasatinib and quercetin have been shown to decrease senescent cell burden and liver damage in mouse models of diet-induced MASLD [100]. These findings highlight the potential of senolytics in mitigating liver diseases’ progression and improving patient outcomes. Despite promising results, translating senolytic therapy to clinical practice for liver diseases presents several challenges. Identifying reliable biomarkers for senescent cell burden, optimizing drug delivery to the liver, and minimizing off-target effects are critical steps. Regarding long-term safety, the potential risk of hepatocellular carcinoma formation due to a reduced immune surveillance demands careful monitoring. Understanding the long-term effects of senolytic treatment and its impact on liver regeneration and function is crucial.

## Conclusions

Inter-organelle communication, particularly through membrane contact sites (MCS), is intrinsic to the cellular efficiency and homeostasis of eukaryotic cells. This complex coordination between organelles, each with its unique environment and function, enables the cell to perform an array of metabolic processes without interference. Among these interactions, the MERCs stand out as a fundamental axis for communication, especially in critical processes such as lipid transfer, Ca^2+^ signaling, protein homeostasis, and the orchestration of cell fate. MERCs play a pivotal role, not only in maintaining the functional integrity of the cellular environment but also in responding to metabolic cues and stress signals. Proteins involved in these tethering sites ensure a highly regulated exchange of signals and materials, highlighting the sophistication of cellular compartmentalization. The dynamic interaction at these contact points influences a host of cellular processes, from apoptosis and autophagy to lipid synthesis and ROS signaling.

Hepatocellular senescence and related liver diseases underscore the significance of these interactions. Senescence, a state of irreversible cell cycle arrest with sustained metabolic activity, is closely linked to disrupted MERCs. This disruption contributes to mitochondrial dysfunction, the accumulation of ROS, and inefficient cellular metabolism, exacerbating conditions such as MASLD, ALD, and viral hepatitis.

Excitingly, senolytics present a promising therapeutic avenue to mitigate the effects of accumulated senescent cells. By restoring MERCs and clearing dysfunctional cells, these drugs may enhance cellular homeostasis and improve organ function. The potential to rejuvenate cellular interactions and ameliorate age-related diseases presents an exciting frontier for future research and therapeutic development.

Future research should focus on developing senolytics with improved specificity for senescent cells at MERCs. Combining senolytics with other therapeutic strategies, such as antifibrotic agents or lifestyle interventions, may enhance their efficacy. Large-scale clinical trials are needed to evaluate the safety and effectiveness of senolytics in diverse populations with liver diseases. Advancements in senolytic therapies could lead to significant improvements in treating age-related liver diseases by targeting the underlying cellular mechanisms contributing to disease progression.

## Data Availability

The materials used in this study can be obtained from the authors by email (pavitra.kumar@charite.de) upon reasonable request.

## Abbreviations

ACLY: ATP citrate lyase
ALD: Alcohol-related liver diseases
ATAD3A: ATPase associated with diverse cellular activities 3A
ATG: Autophagy-related gene
ATF6: Activating transcription factor 6
BAP31: B-cell receptor-associated protein 31
BiP: Binding immunoglobulin protein
CYB5R3: NADH-cytochrome B5 reductase 3
DJ-1: Protein deglycase DJ-1
Drp1: Dynamin-related protein 1
EMRE: MCU-regulating EMRE
ER: Endoplasmic reticulum
ERO1α: ER oxidoreductase 1 alpha
ETC: Electron transport chain
FIS1: Mitochondrial fission 1 protein
FUNDC1: FUN14 domain containing 1
GRP75: Glucose-regulated protein 75
GRP78: Glucose-regulated protein 78 (also known as BiP)
HBV: Hepatitis B virus
HCV: Hepatitis C virus
IMM: Inner mitochondrial membrane
IMS: Intermembrane space
IP3R: Inositol 1,4,5-trisphosphate receptor
IRE1: Inositol-requiring enzyme 1
JNK: C-Jun N-terminal kinase
MAPK: Mitogen-activated protein kinase
MASLD: Metabolic dysfunction-associated steatotic liver disease
MCC: Mitochondrial Ca^2+^ handling complex (implied, not explicitly defined)
MCU: Mitochondrial calcium uniporter
MFN1/2: Mitofusin 1/2
MIM: Mitochondrial import machinery complex
Miro: Mitochondrial Rho GTPase
NAD: Nicotinamide adenine dinucleotide
NADP: Nicotinamide adenine dinucleotide phosphate
NADH: Nicotinamide adenine dinucleotide (reduced)
NF-κB: Nuclear factor kappa-light-chain-enhancer of activated B cells
NOX: Nicotinamide adenine dinucleotide phosphate oxidase
Nrf2: Nuclear factor erythroid 2-related factor 2
OMM: Outer mitochondrial membrane
OPA1: Optic atrophy 1
ORP: Oxysterol-binding protein-related protein
OXPHOS: Oxidative phosphorylation
PACS-2: Phosphofurin acidic cluster sorting protein-2
PC: Phosphatidylcholine
PDZD8: PDZ domain-containing protein 8
PE: Phosphatidylethanolamine
PERK: Protein kinase RNA-like ER kinase
PI3P: Phosphatidylinositol-3-phosphate
PPARα: Peroxisome proliferator-activated receptor alpha
PS: Phosphatidylserine
Psd1p: PtdSer decarboxylase 1
PTPIP51: Protein tyrosine phosphatase interacting protein 51
Rb: Retinoblastoma protein
ROS: Reactive oxygen species
RyR: Ryanodine receptor
SASP: Senescence-associated secretory phenotype
SCP-2: Sterol carrier protein-2
Sig-1R: Sigma-1 receptor
SOD: Superoxide dismutase
SPC: Signal peptidase complex
STX17: Syntaxin 17
Synj2bp: Synaptojanin-2-binding protein
Syx17: Syntaxin 17
TGF-β: Transforming growth factor-beta
TIM: Translocase of the inner mitochondrial membrane
TIS: Therapy-induced senescence
TLR4: Toll-like receptor 4
TOM: Translocase of the outer mitochondrial membrane
UPR: Unfolded protein response
VAPB: Vesicle-associated protein (VAMP)-associated protein B
VDAC: Voltage-dependent anion channel
